# Impacts of Nickel Nanoparticles on Mineral Carbonation

**DOI:** 10.1155/2014/921974

**Published:** 2014-01-22

**Authors:** Marius Bodor, Rafael M. Santos, Yi Wai Chiang, Maria Vlad, Tom Van Gerven

**Affiliations:** ^1^Department of Chemical Engineering, KU Leuven, 3001 Leuven, Belgium; ^2^Department of Environmental Engineering and Metallurgical Technological Systems, “Dunarea de Jos” University of Galati, 800201 Galaţi, Romania; ^3^Department of Microbial and Molecular Systems, KU Leuven, 3001 Leuven, Belgium; ^4^School of Engineering, University of Guelph, Guelph, Canada N1G 2W1

## Abstract

This work presents experimental results regarding the use of pure nickel nanoparticles (NiNP) as a mineral carbonation additive. The aim was to confirm if the catalytic effect of NiNP, which has been reported to increase the dissolution of CO_2_ and the dissociation of carbonic acid in water, is capable of accelerating mineral carbonation processes. The impacts of NiNP on the CO_2_ mineralization by four alkaline materials (pure CaO and MgO, and AOD and CC steelmaking slags), on the product mineralogy, on the particle size distribution, and on the morphology of resulting materials were investigated. NiNP-containing solution was found to reach more acidic pH values upon CO_2_ bubbling, confirming a higher quantity of bicarbonate ions. This effect resulted in acceleration of mineral carbonation in the first fifteen minutes of reaction time when NiNP was present. After this initial stage, however, no benefit of NiNP addition was seen, resulting in very similar carbonation extents after one hour of reaction time. It was also found that increasing solids content decreased the benefit of NiNP, even in the early stages. These results suggest that NiNP has little contribution to mineral carbonation processes when the dissolution of alkaline earth metals is rate limiting.

## 1. Introduction

With a capacity exceeding the needs to sequester all the anthropogenic CO_2_ emissions [1], mineral carbonation represents one of the most promising methods for the mitigation of carbon dioxide originating from industrial sources (e.g., power plants, steel refineries, cement kilns, etc.). However, due to the relatively high stability of natural minerals that could be used in mineral carbonation processes, CO_2_ sequestration through this method is for the moment economically challenging [2]. Therefore, recently, different methods have been studied for the acceleration and/or intensification of carbonates formation through carbon dioxide sequestration in alkaline earth metal rich materials (i.e., containing Ca and Mg—abundant elements that form stable carbonates). The alternative of using natural minerals in mineral carbonation processes is represented by industrial residues usage (e.g., steel slags, incinerator ashes, construction wastes, etc.) [3, 4]. The advantages of using industrial wastes in mineral carbonation processes consist in the high reactivity of these materials compared to natural minerals and also in their positioning near CO_2_ sources, thus reducing transport related costs to a minimum. Although these materials are not available in a sufficient quantity to sequester the entire excess of industrial CO_2_ emissions, they can offer the means to kick-start the meaningful implementation of accelerated CO_2_ mineralization. Simultaneously, the resulting carbonated materials could be valorised for different purposes, thus reducing industrial waste stockpiling. Still, despite being more reactive to carbonation than natural minerals, the industrial wastes also require the development of methods for acceleration and intensification of their mineral carbonation [5]. The methods proposed thus far for this purpose have varied depending on the utilized material, processing route, and usefulness of the resulting material. These methods are most generally split in two main categories: direct and indirect [3, 4], depending on whether CO_2_ reacts in the presence of the alkaline solids, or if the alkaline component are first extracted into solution by lixiviants prior to residual solids separation and CO_2_ introduction; in either case, different adaptations and uses of additives are possible.

Regardless of the method used (indirect or direct carbonation), the simplest option for acceleration and intensification of mineral carbonation is presented by increasing the temperature and/or pressure inside the vessel where the reaction occurs. In this way, Chang et al. [6] obtained one of the best results regarding steelmaking slag (BOF slag) carbonation conversion, 72.2%, by using relatively low temperature and pressure (60°C and 10 bar). Huijgen et al. [7] obtained similar results (conversion = 74%), by using higher temperature and pressure (200°C and 19 bar), for the same type of material. Also, in the case of using steelmaking slag, an extremely important parameter is the size of component particles; to obtain fine particles, ideal for high carbonation reactivity, a high quantity of milling/grinding energy is needed. To overcome this, Santos et al. [8] suggested that the cooling rates and the implicit composition of steelmaking slag should be optimized in such a way as to obtain a material that can react more readily with CO_2_. Another proposed method consists in performing mineral carbonation during BOF slag hot-stage granulation; in this way the milling step would be eliminated and the intrinsic heat (released from the molten slag during cooling) would be used to aid the mineral carbonation process [9]. Other possibilities for acceleration and/or intensification of mineral carbonation reactions have been proposed by different research groups, consisting in using additives like acetic acid (CH_3_COOH) [10], nitric acid (HNO_3_), sulphuric acid (H_2_SO_4_), sodium chloride (NaCl), and diverse ammonium salts (NH_4_Cl, NH_4_NO_3_, NH_4_H_2_PO_4_, (NH_4_)_2_HPO_4_, and (NH_2_)_2_CO) [11]. All these, whilst presenting promising results, are still far from implementation at an industrial scale due to high costs, process complexities, optimization, and regeneration needs.

The work presented in this paper is related to two earlier papers of our research group [12, 13], in that similar mineral carbonation processes are being used (mineral carbonation at low temperature, atmospheric pressure, and applying mechanical or ultrasound mixing). Herein, experiments are performed using pure calcium and magnesium oxides and steelmaking slags (argon oxygen decarburization (AOD) and continuous casting (CC)). Compared to the experiments from the previous works, the objective of the present study was to test the use of a novel additive (pure nickel nanoparticles (NiNP)) applied in very low quantities, namely, 30 ppm (i.e., 30 mg/L). According to Bhaduri and Šiller [14], nickel nanoparticles increase the solubility of CO_2_ in water (up to an optimal additive concentration of 30 ppm) and can promote the formation of bicarbonate ions (HCO_3_^−^) in solution, which can then participate in the carbonation reaction of alkaline earth metals (such as Ca and Mg). The mechanism of this reaction, illustrated by (1)–(3), offers details regarding the means by which the dissolved CO_2_ quantity is increased in water. This happens through nanoparticle interaction with water molecules; hydroxyl groups (OH^−^) are thought to be formed on the particle's surface, which in turn react with CO_2_ to form bicarbonate ions. This mechanism is similar to the one in which a zinc containing enzyme is used (bovine carbonic anhydrase) [15], but the advantage of using pure nickel nanoparticles consists in their susceptibility to being magnetically recovered from the solution and reutilized and to its lower cost compared with the enzyme:

(1) $$\begin{array}{c} \text{NiNP-}{\text{H}}_{2}\text{O}⟶\text{NiNP-}{\text{OH}}^{-}+{\text{H}}^{+} \end{array}$$

(2) $$\begin{array}{c} \text{NiNP-}{\text{OH}}^{-}+{\text{CO}}_{2}⟶\text{NiNP-H}{{\text{CO}}_{3}}^{-} \end{array}$$

(3) $$\begin{array}{c} \text{NiNP-H}{{\text{CO}}_{3}}^{-}+{\text{H}}_{2}\text{O}⟶\text{NiNP-}{\text{H}}_{2}\text{O}+\text{H}{{\text{CO}}_{3}}^{-\;} \end{array}$$

The study of Bhaduri and Šiller [14] did not, however, test the effect of these nanoparticles on mineral carbonation kinetics or conversion. The purpose of the experiments conducted in the present study aimed to confirm if the mineral carbonation process of alkaline earth metal rich materials can be accelerated by using pure nickel nanoparticles as a catalytic additive. The obtained carbonated materials were analyzed from a mineralogical point of view to establish the variation of carbonate quantity and composition and from a morphological point of view to analyze the physical structure evolution (particle size, shape, and surface texture) after the mineral carbonation process.

## 2. Materials and Methods

### 2.1. Alkaline Materials

Carbonation experiments were conducted using four types of materials with different contents of alkaline earth metals. Analytical grade burnt lime (hereafter referred to as CaO), with calcium oxide content of 98.3 wt%, and analytical grade magnesia (hereafter referred to as MgO), with magnesium oxide content of 99.0 wt%, were obtained from Chem-Lab. Argon oxygen decarburization (AOD) and continuous casting (CC) steelmaking slags, with calcium oxide contents of 56.8 wt% and 52.0 wt%, respectively, were obtained from a stainless steel producer and sieved to <500 *μ*m to remove coarse particles. The full chemical and mineralogical compositions of these materials are presented in Tables 1 and 2, respectively.

### 2.2. Carbonation Tests

Slurry carbonation experiments were conducted using a laboratory glass beaker with a volume of two litres and diameter of approximately 14 cm. The suspension was mixed solely by a mechanical stirrer (Heidolph type RZ-R1) with straight blade impeller at 340 rpm for stirred experiments or by an ultrasound horn during sonication experiments. The ultrasound horn consisted of a Hielscher UP200S processor, which operates at 24 kHz frequency and delivers 200 W gross power, coupled to an S14 sonotrode, which has a tip diameter of 14 mm, maximal amplitude of 125 *μ*m, and an acoustic power density of 105 W/cm². A PT100 temperature sensor was used to monitor solution temperature.

Carbonation experiments were performed either with 10 g of material (or the CaO molar equivalent of 7.19 g for MgO experiments) or with 50 g of material (or the CaO molar equivalent of 35.95 g for MgO experiments), added to one litre of ultrapure water, which reached a height of 7.5 cm in the beaker; for sonication experiments the probe tip was immersed 3.5 cm from the beaker bottom. For experiments containing NiNP, this additive, obtained from Sigma-Aldrich (≥99% Ni, <100 nm), was added in quantities ranging from 30 to 300 mg. Temperature was controlled by the use of a hot plate (IKAMAG RCT) for heating (in the case of stirred experiments) and water bath for cooling (in the case of sonicated experiments, since ultrasound produces heat, which must be dissipated to maintain a constant temperature). A temperature of 50°C ± 2°C was maintained during carbonation experiments. Carbon dioxide addition commenced from the beginning of the experiment, delivered to the solution by bubbling from a compressed gas cylinder (≥99.5% purity), with flow controlled at 0.72 NL/min by a Brooks Sho-rate rotameter; NiNP was added at the same time as CO_2_, while alkaline solids were added once the solution reached the desired temperature. Carbonation duration varied from fifteen to sixty minutes, after which the slurry was filtered (Whatman number 2 filter paper), and the recovered solids were dried at 105°C overnight.

In addition to performing the mineral carbonation experiments, the evolution of the pH value of the solution as a function of time, during CO_2_ bubbling in absence of alkaline solids, was also measured. To investigate the influence of NiNP addition to the solution, continuous measurement of pH value was performed during 22 minutes using one litre solutions of ultrapure water and ultrapure water containing 30 mg NiNP. Measurement of pH started after the solution reached 50°C temperature and once CO_2_ bubbling began.

### 2.3. Analytical Methods

Chemical composition of solid samples was determined by X-ray fluorescence (XRF, Panalytical PW2400). Mineralogical composition was determined by X-Ray diffraction (XRD), performed on a Philips PW1830 equipped with a graphite monochromator and a gas proportional detector, using Cu K*α* radiation at 30 mA and 45 kV, step size of 0.03° 2*θ*, and counting time of 2 s per step, over 10–65° 2*θ* range. Mineral identification was done in Diffrac-Plus EVA (Bruker) and mineral quantification (QXRD) was performed by Rietveld refinement technique using Topas Academic v4.1 (Coelho Software). The volume-based particle size distributions and average particle diameters were determined by wet laser diffraction (LD, Malvern Mastersizer S). The powder morphology was observed by scanning electron microscopy (SEM, Philips XL30). The CO_2_ uptake of carbonated CaO and MgO was determined by QXRD. The quantified phases included aragonite, calcite, dolomite, huntite, mono- and ortho-hydromagnesite, magnesian calcite, magnesite, monohydrocalcite, nesquehonite, spurrite, and vaterite. It was verified using standard mixtures of pure CaCO_3_ and pure Ca(OH)_2_ that carbonate quantification accuracy was ±1.6 wt%. The CO_2_ uptake of carbonated slags, whose complex mineralogy makes XRD quantification less accurate, was quantified by thermal gravimetric analysis (TGA, Netzsch STA 409), operated from 25 to 900°C under nitrogen flow at a heating rate of 15°C/min. The amount of CO_2_ released was quantified by the weight loss between 400 and 900°C, which is attributable to carbonate decomposition.

## 3. Results and Discussion

### 3.1. Influence of NiNP on the Solution's pH Value

**Figure 1:**
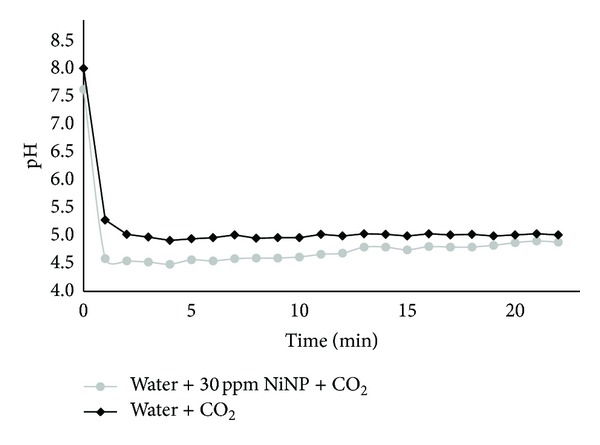
Evolution of pH value for ultrapure water with/without NiNP addition, after commencement of CO_2_ bubbling at 50°C.

Figure 1 presents the pH value versus time evolutions for two solutions tested, one containing ultrapure water only and another containing 30 ppm NiNP. It can be observed that once CO_2_ bubbling commences, the pH of both solutions drops rapidly. The pH of the NiNP containing solution reaches lower pH values, by approximately 0.43 pH units in the first five minutes. This difference, at the pH range in question (~4.5 to 5), is equivalent to an extra 0.018 mM H^+^ in the NiNP solution, which means 2.7 times greater [H^+^] than in the pure water solution. This extra H^+^ can be directly linked to extra HCO_3_^−^ and CO_3_^2−^ forming in the NiNP solution. Bhaduri and Šiller [14] pointed to a three-time increase in CO_2_ solubility (total, not just dissociated) in water containing 30 ppm NiNP, so a similar factor to the 2.7 herein estimated for increased carbonic acid dissociation.

The pH of the NiNP solution remains lower for the 22-minute duration of the experiment, though the difference between the curves decreases over time. Bhaduri et al. [16] observed that in equilibrium studies the reaction rate of the reaction between carbonic acid and sodium carbonate is not catalysed by NiNP, but when the same reaction is done in a nonequilibrium system (e.g., with alkaline pH due to 0.1 M sodium carbonate addition) the enhancement in rate is obtained. This may explain the diminishing effect of NiNP over time as the solution equilibrates. The herein obtained nonequilibrium data are in accordance with those presented by Bhaduri and Šiller [14] and suggest the presence of carbonic acid (H_2_CO_3_) and the formation of (bi) carbonate ions to be higher in case of NiNP application. Higher dissolved CO_2_ concentrations may be beneficial for acceleration of mineral carbonation reactions. The results obtained from mineral carbonation experiments, with and without NiNP addition, are presented next.

### 3.2. CO_2_ Uptake Following Mineral Carbonation Process

The experiments have been split in two series; firstly, pure calcium and magnesium oxides were utilized for establishing the optimal parameters, and subsequently steelmaking slags (AOD and CC) were used. First trials were done by using a solid/liquid (S/L) ratio of 10 g CaO/1000 mL ultrapure water, or the equivalent in moles for MgO, namely, 7.19 g MgO/1000 mL ultrapure water. However, for this S/L ratio, the results showed an almost maximum conversion of CaO regardless the reaction time (15, 30, or 60 minutes). A reason for this phenomenon is represented by CO_2_ bubbling in the solution during the entire period needed to reach 50°C temperature (maintained afterwards constant throughout the experiment). The amount of dissolved CO_2_ in the water prior to solids addition (19.6 mM, according to Visual MINTEQ (ver. 3.0 beta) geochemical modelling) plus that bubbled during the first 15 minutes (441 mM at 0.72 NL/min) was sufficient to carbonate the amount of solids added (179 mM). This means that at low solids loading, no detectable (for practical reasons 15 minutes deemed the shortest possible experimental duration) benefit can be derived from the addition of NiNP for CaO carbonation. As such, the subsequent trials were done using a five-time larger S/L ratio, namely, 50 g/1000 mL for CaO or the molar equivalent (893 mM) of 35.95 g/1000 mL for MgO. This same ratio was also used for the steelmaking slags mineral carbonation experiments, presented later, so that an efficient comparison could be realized between data from all materials used in carbonation experiments.

**Figure 2:**
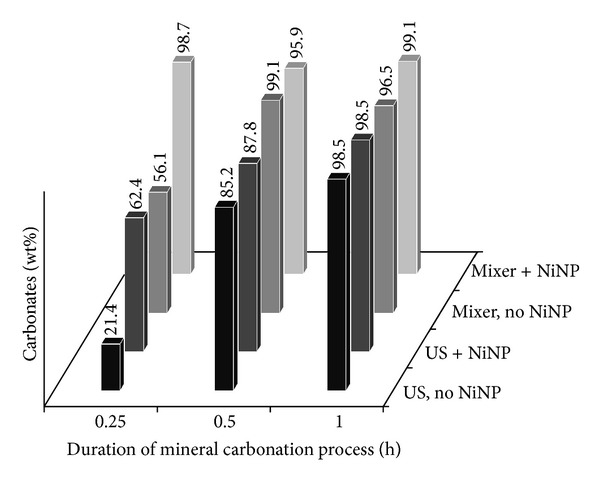
Carbonate content after MgO mineral carbonation, using S/L ratio of 7.19 g/1000 mL, determined by QXRD.

Data obtained using the lower S/L ratio for the case of MgO carbonation are presented in Figure 2. These data demonstrate the contribution of NiNP to the acceleration of mineral carbonation process. Process acceleration is emphasized through higher quantities of carbonates obtained in the first 15 minutes (21.4 wt% by using ultrasound for mixing and 62.4 wt% when ultrasound is used along with NiNP addition, and 56.1 wt% when mechanical stirrer is used for mixing and 98.7 wt% when stirrer and NiNP are used together). Results using ultrasound instead of the mechanical stirrer were inferior due to poor mixing of the viscous MgO slurry by sonication (unlike in the case of CaO where mixing by both methods is satisfactory). After 30 minutes of reaction duration these discrepancies become less obvious, and differences among samples fall within the estimated quantification error by Rietveld refinement of ±1.6 wt%. These trends suggest that a longer duration for mineral carbonation process can compensate for the lack of NiNP addition. So, it can be said that less time is needed to reach a certain level of carbonation when NiNP addition is employed.

**Figure 3:**
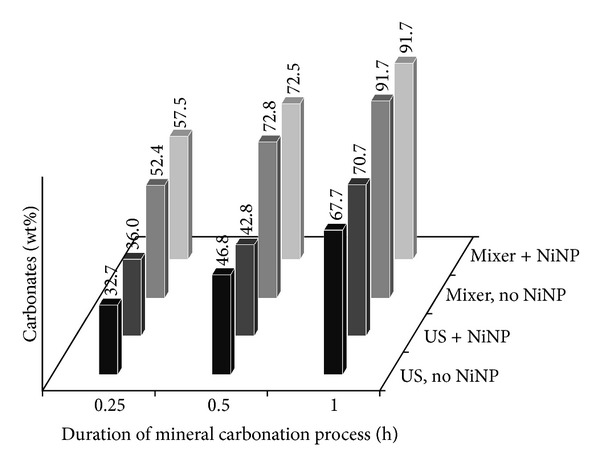
Carbonate content after CaO mineral carbonation, using S/L ratio of 50 g/1000 mL, determined by QXRD.

Figure 3 shows data when calcium oxide was used, however, this time with the S/L ratio being higher (50 g/1000 mL). Due to an increase in quantity of the material used for experiments, calcium oxide conversion suffered a slowdown, this being highlighted by the lower values of obtained carbonates, even after one hour of carbonation. Compared to the case when a lower S/L ratio was used and the conversion approached the maximum value even after 15 minutes, this time high conversion levels (91.7 wt%) were only obtained after one hour of carbonation under mechanical mixing, with or without NiNP addition. It is thought that carbonation conversion under ultrasound mixing was hindered due to poor mixing of the high solids loading used here (50 g/L), compared to the results of Santos et al. [13] where 10 g/L was agitated by a combination of mechanical and ultrasound mixing (a lower CO_2_ flow rate of 0.24 NL/min was also therein used). At the early stages of carbonation, the carbonate contents of samples prepared with NiNP were slightly higher than those prepared in pure water. However, the benefit of NiNP addition was reduced compared to the case of MgO, in that the average augmentation of carbonate content in the CaO samples was only 4.2 wt%, compared to 41.8 wt% for the MgO samples. Increasing the concentration of NiNP to 300 ppm was also attempted to ensure that the amounts used are not too small, though Bhaduri and Šiller [14] indicated that such high concentrations are unlikely to be beneficial. In the present case, it was found that increasing NiNP concentration does not bring about significant benefit, with carbonate content slightly decreasing to 51.9 wt% after 15 minutes and slightly increasing to 75.0 and 97.2 wt% after 30 and 60 minutes, respectively.

**Figure 4:**
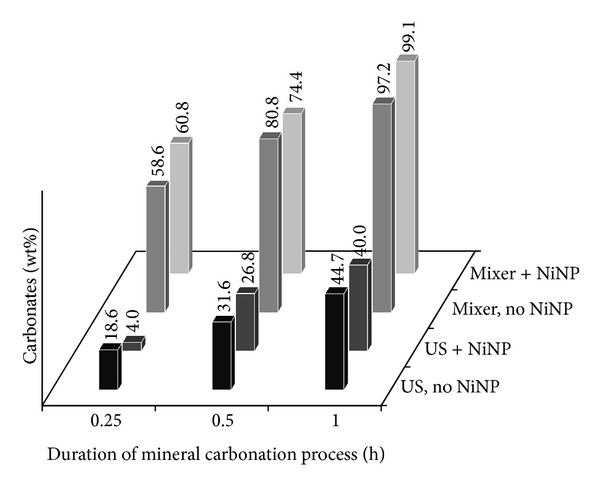
Carbonate content after MgO mineral carbonation, using S/L ratio of 35.95 g/1000 mL, determined by QXRD.

When the concentration of MgO is increased from 10 to 50 g/L, the benefit of NiNP addition with mechanical mixing is much reduced in the first 15 minutes (Figure 4) and essentially disappears for longer reaction times. For the case of ultrasound mixing, where the CO_2_ uptake becomes slowed again due to poor mixing, no benefit of NiNP is detected. It thus appears that the increased initial solubility of CO_2_ catalyzed by NiNP is largely responsible for the increased carbonation early in the reaction phase. However, when the solids content increases, the contribution of the extra dissolved CO_2_ diminishes, to the point that at high solids loadings the CO_2_ supply rate and alkaline earth metal dissolution rate become rate limiting. As such, NiNP addition would appear to be most interesting for dilute systems, where the concentration of Ca or Mg in the solution is closer to the order of magnitude of CO_2_ solubility, or for systems where the alkaline earth metals are already solubilised and thus readily available for reaction from the start. This type of situation is more likely found during the carbonation of brines and brackish waters [17, 18] than in mineral carbonation systems.

To confirm the aforementioned effects of NiNP, further mineral carbonation experiments were performed using AOD and CC steelmaking slags, as these materials are considered some of the most readily available and reactive (due to powdery morphology and high calcium silicate content) for industrial mineral carbonation implementation [8]. Based on the results from pure oxides carbonation experiments, under the conditions herein tested, it was concluded that mechanical mixing is more efficient than ultrasound mixing. Therefore mineral carbonation experiments on steelmaking slags were carried out using mechanical mixing.

**Figure 5:**
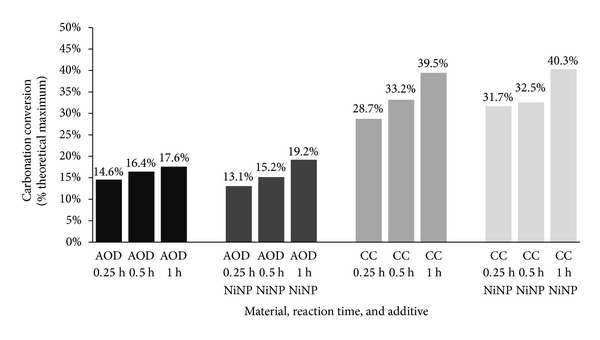
Carbonation conversion of AOD and CC slags after mineral carbonation, using S/L ratio of 50 g/1000 mL, determined by TGA.

Results obtained for CC slag (Figure 5) present similar trend to the ones from CaO and MgO carbonation experiments, namely, a greater conversion in the first 15 minutes when NiNP addition was used; however, after 30 minutes this effect is no longer present. For AOD slag, no benefit of NiNP addition is obtained. Moreover, AOD generally carbonated much less than CC slag, in accordance with previous studies [8, 13]. Seeing as only CC slag benefited from NiNP addition, it can be said that only those materials that carbonate rapidly can benefit from NiNP addition in the early phases of the reaction, since when carbonation is slow, the rate limiting step is from the very beginning the dissolution of alkaline earth metals from the mineral matrix, rather than CO_2_ availability in solution (the benefit that NiNP brings). Thus, as stated earlier, mineral carbonation does not appear to benefit significantly from the addition of NiNP from the point of view of carbonation kinetics and extent. Next, it is investigated if NiNP has any effect on the mineralogy and morphology of carbonated materials.

### 3.3. Influence of NiNP on Mineralogical Composition of Carbonated Materials

The use of additives during mineral carbonation can have an influence on the types of carbonate phases formed. For example, in our previous studies [12, 19] it was found that the self-regenerative additive magnesium chloride (MgCl_2_) promotes the formation of the aragonite polymorph of calcium carbonate, and it can also induce the greater presence of magnesian calcite. Magnesium chloride participates in the mineral carbonation reaction by controlling calcium dissolution from alkaline materials, calcium precipitation from solution, and carbonate crystal nucleation and growth. Thus, it does not interfere with the carbonic acid formation and dissociation processes, as is the case with NiNP. As such, it is of interest to study if NiNP can have an influence on the carbonate mineral phases formed, besides affecting the reaction kinetics. Here, the type and quantity of the mineral phases obtained when carbonation was done with and without the use of NiNP was assessed by QXRD. Results for CaO and MgO experiments are presented in Table 3.

Table 3 presents the mineralogical composition of the fifteen-minute and one-hour carbonated calcium and magnesium oxides, with and without NiNP addition, and prepared with mechanical mixing. No significant differences can be observed between results obtained by carbonation with or without NiNP addition, except that, as presented earlier, the total amount of carbonates is slightly higher in case of NiNP utilization. Calcite is the predominant carbonate formed from CaO, while hydromagnesite is the predominant phase formed from MgO, regardless of NiNP addition.

One mineralogical effect that is clearly discernible is the formation of portlandite and brucite (hydroxides of Ca and Mg, resp.) early in the reaction, due to hydration of the oxides, which then go on to become carbonated. Also, from CaO derived samples, it can be seen that more hydrocalcite and vaterite are present early in the reaction, while more aragonite forms at the extended reaction time. Hydrocalcite and vaterite are known to be metastable forms of calcium carbonates [8, 20], which can explain their transient behaviour. In the case of MgO derived samples, magnesite and nesquehonite only form in small quantities (0.9–2.2 wt%) at the longer reaction duration. These carbonates are known to form at more intensive carbonation conditions, while hydromagnesite is preferably formed at higher pH [8], hence hydromagnesite being the predominant carbonate formed in the present experiments.

The same small differences of minerals type and quantities were obtained for the steelmaking slags (not shown). It can be thus concluded that NiNP addition has no great effect on mineralogical distribution of the carbonate phases in carbonated materials. As aforementioned, this is likely related to the fact that NiNP affects carbonic acid formation and dissociation but does not affect the dissolution or precipitation of alkaline earth metal cations.

### 3.4. Influence of NiNP on Particle Size Distribution of Carbonated Materials

Another parameter studied, regarding the influence of NiNP addition to mineral carbonation experiments, was the particle size distribution of the obtained carbonate precipitates. This analysis is of interest since carbonation additives can have an effect on the crystallization nucleation and growth rate and thus on the size of the individual precipitate particles (these effects are exemplified in the works of Santos et al. [12, 19]). In the case of NiNP, the accelerated early carbonation discussed previously could have the potential to induce greater nucleation rate and thus particle size reduction or conversely accelerated crystal growth and thus particle size increase. That is, any significant deviation in particle size distribution would warrant further investigation into the mechanism. Results of laser diffraction measurements are presented in Table 4 and consist in the volume moment mean diameter (*D*[4,3]) and surface area moment mean diameter (*D*[3,2]) of CaO and MgO derived precipitates after one-hour carbonation under mechanical stirring, with and without NiNP addition.

As in the mineralogical analysis, no significant difference is found between samples prepared in the presence of NiNP and those prepared in pure water. In the case of CaO there is a slight decrease of average particle size with NiNP addition, whereas there is a slight increase in the case of MgO; these variations are within expected experimental and analytical deviation. As such, it can be said that there is a lack of influence of nickel nanoparticle addition on the particle size distribution of carbonate crystal precipitates. In both cases the particle size histograms (not shown) simply shift slightly but retain very similar shapes; carbonated CaO has a bimodal distribution, with a smaller mode between 0.1 and 1 *μ*m and a larger mode between 4 and 40 *μ*m (both nearly symmetrical), while carbonated MgO has an asymmetrical (skewed to larger sizes) unimodal distribution between 0.8 and 150 *μ*m. These size distributions are analogous to those of the raw materials (i.e., CaO and MgO), but the sizes are significantly smaller (*D*[4,3] of CaO being 41.9 *μ*m and that of MgO being 129.1 *μ*m), which suggests that the formed carbonates crystallize into newly formed particles rather than onto the original particles.

### 3.5. Influence of NiNP on Particle Morphology of Carbonated Materials

**Figure 6:**
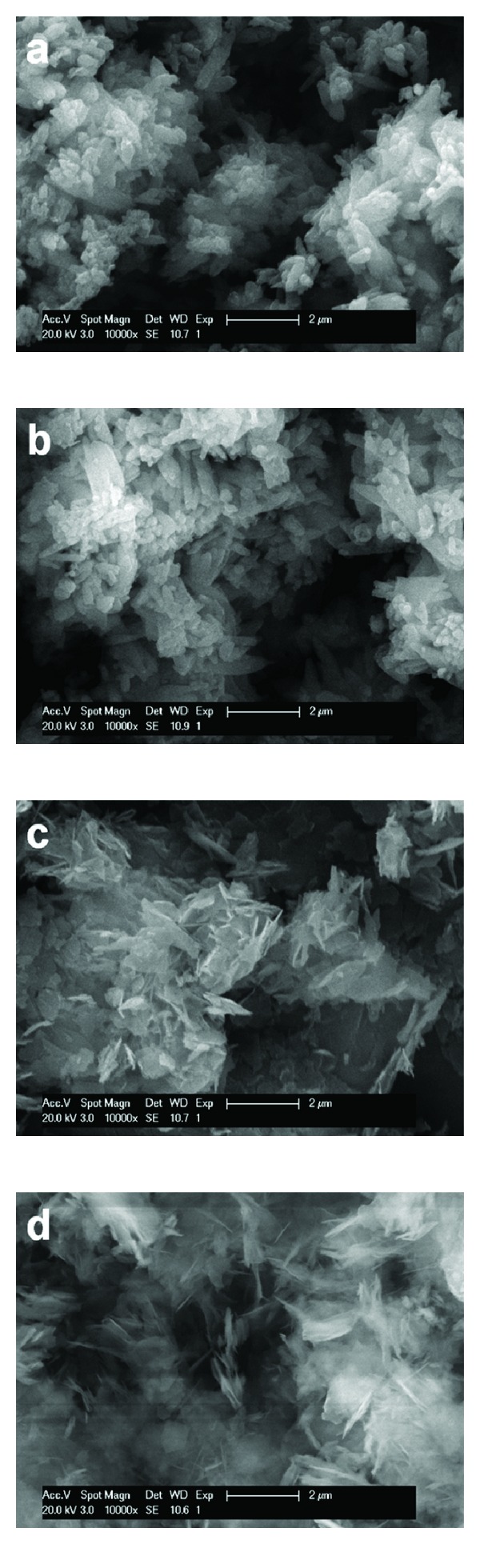
Morphology of CaO, carbonated for one hour, at 50°C and 50 g/L, with mechanical stirring, without NiNP addition (a) and with NiNP addition (b); morphology of MgO, carbonated with the same parameters except solids loading of 35.95 g/L, without NiNP addition (c) and with NiNP addition (d), realized by SEM imaging.

**Figure 7:**
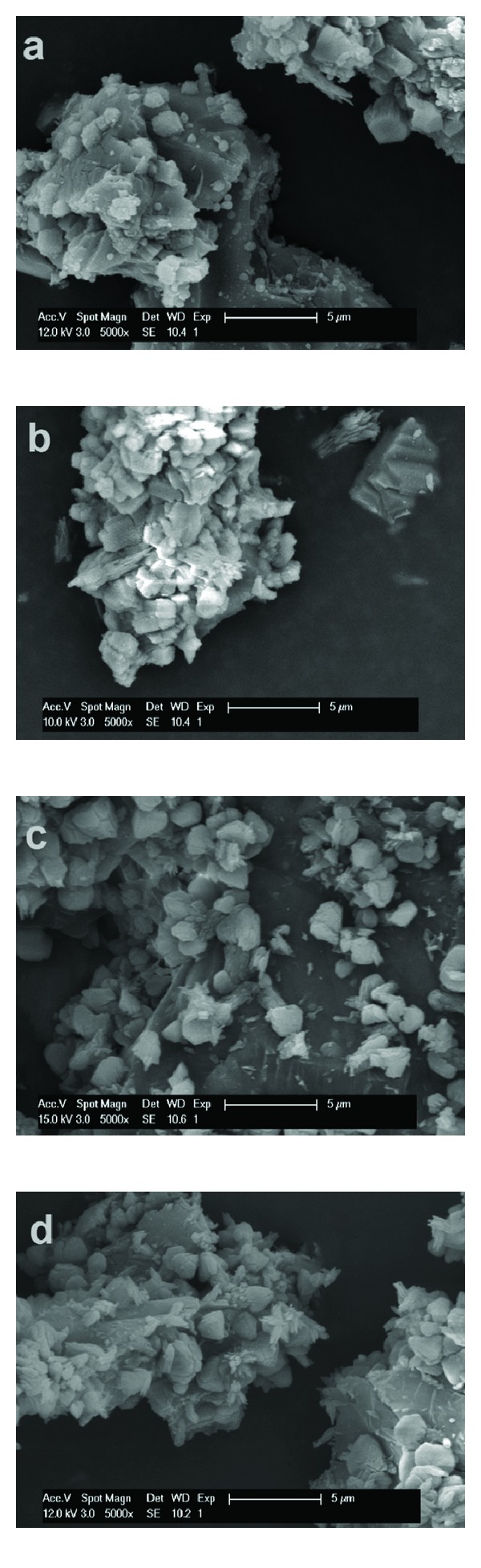
Morphology of AOD slag, carbonated for one hour, at 50°C and 50 g/L, with mechanical stirring, without NiNP addition (a) and with NiNP addition (b); morphology of CC slag, carbonated with the same parameters, without NiNP addition (c) and with NiNP addition (d), realized by SEM imaging.

Studying the morphology of carbonated materials, with or without NiNP addition, by SEM, highlighted the formation of carbonates by precipitation as newly formed particles (in the case of CaO and MgO derived materials, see Figure 6) or on the surface of the constituent particles of the raw material (in the case of carbonated steelmaking slags, see Figure 7). Figure 6 presents the comparison between particle morphology of CaO (Figures 6(a) and 6(b)) and MgO (Figures 6(c) and 6(d)), after one-hour long mineral carbonation process, at 50°C and high solids loading, mechanical stirring, without NiNP addition (Figures 6(a) and 6(c)) and with NiNP addition (Figures 6(b) and 6(d)). Figure 7 presents the comparison between particle morphology of AOD slag (Figures 7(a) and 7(b)) and CC slag (Figures 7(c) and 7(d)), after one-hour long mineral carbonation process, 50°C and 50 g/L, mechanical stirring, without NiNP addition (Figures 7(a) and 7(c)) and with NiNP addition (Figures 7(b) and 7(d)).

In all cases, the carbonated materials presented the same crystal morphology irrespective of NiNP addition, which confirms the observations previously presented from mineralogical and laser diffraction analyses. Carbonated CaO particles resembled typical scalenohedral calcite crystals, while carbonated MgO particles resembled typical platy hydromagnesite crystals (Figure 6). Furthermore, in the case of AOD and CC slags, the results regarding carbonated particle morphology (Figure 7) are in concordance with the analyses performed by Santos et al. [12, 13], respectively. The resembling structures observed through SEM analysis prove that using pure nickel nanoparticles as an additive during mineral carbonation has no effect on the morphology of the carbonated materials. Thus, based on this observation and the results on the early stage acceleration of carbonate formation and the solution acidification, the statement according to which NiNP addition has only an effect of increasing the CO_2_ dissolution and dissociation in the aqueous solution is supported.

## 4. Conclusions

Carbon dioxide mitigation through mineral carbonation is still economically inefficient and thus not yet fit for implementation on an industrial scale, despite the mounting knowledge pool on the subject. This inefficiency is due to high energy demand, slow reaction kinetics, and low conversion degrees (and implicitly limited CO_2_ sequestration). For this reason, in the last years, different methods have been proposed for mineral carbonation acceleration and efficiency purposes. Additive usage remains an interesting choice to accelerate CO_2_ uptake, especially if mild process conditions can be maintained. To this end, most researchers have sought additives that increase mineral dissolution and carbonate precipitation. In another study of this research group [12], magnesium chloride addition was used with the purpose of augmenting calcium dissolution from several alkaline materials suitable for ex situ mineral carbonation, thus intensifying the process. The additive used in the present study, pure nickel nanoparticles (NiNP), has another purpose, namely, increasing dissolved CO_2_ dissolution and dissociation in the aqueous solution, as divulged by Bhaduri and Šiller [14], conceptualizers of this approach, and thereby potentially accelerating the CO_2_ uptake rate by the carbonating material, herein tested.

The confirmation of dissolving a higher quantity of CO_2_ in the NiNP-containing solution was done by studying the evolution of solution's pH value; more acidic values obtained in the presence of NiNP suggest an increased presence of dissociated carbonic acid. This higher quantity of dissolved CO_2_ resulted in acceleration of the mineral carbonation process in the first fifteen minutes of reaction time when NiNP was present. This was demonstrated by the results regarding the obtained carbonate quantities, determined by QXRD and TGA, using pure CaO and MgO materials, as well as one of the two steelmaking slags tested (CC slag). After this initial stage, however, no further benefit of NiNP addition was seen, resulting in very similar carbonation extents after one hour of reaction time. This suggests that NiNP has little contribution to the carbonation rate when the dissolution of alkaline earth metals is rate limiting, rather than the CO_2_ solubility or carbonate/bicarbonate ion concentrations. It was also found that increasing solids content decreased the benefit of NiNP even in the early stages, since the fraction of extra dissolved CO_2_ to the required amount of CO_2_ for carbonation decreases with higher solids loading. Likewise, the presence of NiNP did not result in significant differences regarding carbonate mineral composition nor particle and crystal morphologies. This is unlike the effect MgCl_2_ had in our prior studies, which suggests that NiNP does not interfere in the carbonation precipitation processes such as nucleation and crystal growth.

To further accelerate the NiNP-assisted process, an approach could be represented by reducing the loading of alkaline solids used, in order to avoid the CO_2_ supply rate or the alkaline earth metal dissolution rate from becoming the rate determining step. Alternatively, carbonation of solutions containing dissolved alkaline earth metals, such as brines and brackish waters, may benefit more from NiNP addition than mineral carbonation systems.

## Figures and Tables

**Table 1:** Chemical composition (expressed as wt% oxides) of alkaline materials determined by XRF (normalized to 100 wt% total; ≥1.0 wt% shown).

	Al_2_O_3_	CaO	Cl	Fe_2_O_3_	K_2_O	MgO	MnO	Na_2_O	SO_3_	SiO_2_	TiO_2_
CaO	<	98.3	<	<	<	1.1	<	<	<	<	<
MgO	<	<	<	<	<	99.0	<	<	<	<	<
AOD slag	1.0	56.8	<	<	<	7.5	<	<	<	32.5	<
CC slag	1.1	52.0	<	1.3	<	9.9	<	<	<	27.5	<

<: less than 1.0 wt%.

**Table 2:** Mineral composition of alkaline materials determined by QXRD (wt% of crystalline total).

Mineral name	Chemical formula	CaO	MgO	AOD slag	CC slag
Åkermanite	Ca_2_MgSi_2_O_7_	nd	nd	1.9	0.1
Bredigite	Ca_7_Mg(SiO_4_)_4_	nd	nd	22.1	11.3
Brucite	Mg(OH)_2_	nd	32.5	0.1	nd
Cuspidine	Ca_4_Si_2_O_7_F_2_	nd	nd	11.3	9.3
*β*-Dicalcium silicate	Ca_2_SiO_4_	nd	nd	6.4	3.6
*γ*-Dicalcium silicate	Ca_2_SiO_4_	nd	nd	27.9	40.1
Fayalite	Fe_2_SiO_4_	nd	nd	0.2	1.0
Enstatite	MgSiO_3_	nd	nd	4.3	11.4
Gehlenite	Ca_2_Al_2_SiO_7_	nd	nd	0.6	1.7
Lime	CaO	88.8	nd	nd	nd
Magnetite	Fe_3_O_4_	nd	nd	0.6	1.9
Merwinite	Ca_3_Mg(SiO_4_)_2_	nd	nd	14.6	3.3
Periclase	MgO	nd	67.5	7.4	10.7
Portlandite	Ca(OH)_2_	11.2	nd	0.5	2.1
Quartz	SiO_2_	nd	nd	0.6	0.9
Wollastonite	CaSiO_3_	nd	nd	1.3	2.5

nd: not detected (<0.1 wt%).

**Table 3:** Mineral composition of CaO and MgO after fifteen-minute and one-hour carbonation experiments (S/L = 50 g/L), under mechanical mixing, with/without NiNP addition, determined by QXRD (wt% of crystalline total).

Mineral name	Chemical formula	CaO		MgO	
		No NiNP	+NiNP	No NiNP	+NiNP
		0.25/1 h	0.25/1 h	0.25/1 h	0.25/1 h
Aragonite	CaCO_3_	0.8/12.8	0.5/8.0	nd	nd
Brucite	Mg(OH)_2_	nd	nd	38.2/2.0	36.1/0.8
Calcite	CaCO_3_	46.7/78.2	51.7/81.6	nd	nd
Hydromagnesite^1^	Mg_5_(CO_3_)_4_(OH)_2_·4(H_2_O)	nd	nd	58.5/95.7	60.6/96.1
Lime	CaO	1.6/0.4	1.4/0.3	nd	nd
Magnesite	MgCO_3_	nd	nd	nd/1.5	0.1/0.9
Monohydrocalcite	CaCO_3_·(H_2_O)	2.6/0.2	1.5/1.2	nd	nd
Nesquehonite	Mg(HCO_3_)(OH)·2(H_2_O)	nd	nd	0.1/nd	0.2/2.2
Periclase	MgO	nd	nd	3.2/0.8	3.1/0.1
Portlandite	Ca(OH)_2_	46.0/7.9	41.1/8.0	nd	nd
Vaterite	CaCO_3_	2.4/0.5	3.7/0.9	nd	nd

nd: not detected (<0.1 wt%).

^1^sum of mono- and ortho-hydromagnesite.

**Table 4:** Average particle size diameters, expressed as volume moment mean diameter (*D*[4,3]) and surface area moment mean diameter (*D*[3,2]) of alkaline materials after one-hour carbonation experiments (S/L = 50 g/L), under mechanical mixing, with/without NiNP addition, determined by LD (all values expressed in µm).

Carbonated material	*D*[4,3]	*D*[4,3]	*D*[3,2]	*D*[3,2]
	No NiNP	+NiNP	No NiNP	+NiNP
CaO	10.8	10.2	1.3	1.1
MgO	22.3	26.1	10.8	11.6

## References

[1] Sipilä J, Teir S, Zevenhoven R (2008). *Carbon Dioxide Sequestration by Mineral Carbonation Literature Review Update 2005-2007*.

[2] Hitch M, Dipple GM (2012). Economic feasibility and sensitivity analysis of integrating industrial-scale mineral carbonation into mining operations. *Minerals Engineering*.

[3] Bodor M, Santos RM, Van Gerven T, Vlad M (2013). Recent developments and perspectives on the treatment of industrial wastes by mineral carbonation—a review. *Central European Journal of Engineering*.

[4] Bobicki ER, Liu Q, Xu Z, Zeng H (2012). Carbon capture and storage using alkaline industrial wastes. *Progress in Energy and Combustion Science*.

[5] Santos RM, Van Gerven T (2011). Process intensification routes for mineral carbonation. *Greenhouse Gases*.

[6] Chang E-E, Chen C-H, Chen Y-H, Pan S-Y, Chiang P-C (2011). Performance evaluation for carbonation of steel-making slags in a slurry reactor. *Journal of Hazardous Materials*.

[7] Huijgen WJJ, Witkamp G-J, Comans RNJ (2005). Mineral CO_2_ sequestration by steel slag carbonation. *Environmental Science and Technology*.

[8] Santos RM, van Bouwel J, Vandevelde E, Mertens G, Elsen J, Van Gerven T (2013). Accelerated mineral carbonation of stainless steel slags for CO_2_ storage and waste valorization: effect of process parameters on geochemical properties. *International Journal of Greenhouse Gas Control*.

[9] Santos RM, Ling D, Sarvaramini A (2012). Stabilization of basic oxygen furnace slag by hot-stage carbonation treatment. *Chemical Engineering Journal*.

[10] Eloneva S, Teir S, Salminen J, Fogelholm C-J, Zevenhoven R (2008). Steel converter slag as a raw material for precipitation of pure calcium carbonate. *Industrial and Engineering Chemistry Research*.

[11] Eloneva S, Teir S, Revitzer H (2009). Reduction of CO_2_ emissions from steel plants by using steelmaking slags for production of marketable calcium carbonate. *Steel Research International*.

[12] Santos RM, Bodor M, Dragomir PN, Vraciu AG, Vlad M, Van Gerven T (2013). Magnesium chloride as a leaching and aragonite-promoting self-regenerative additive for the mineral carbonation of calcium-rich materials. *Minerals Engineering*.

[13] Santos RM, François D, Mertens G, Elsen J, Van Gerven T (2013). Ultrasound-intensified mineral carbonation. *Applied Thermal Engineering*.

[14] Bhaduri GA, Šiller L (2013). Nickel nanoparticles catalyse reversible hydration of carbon dioxide for mineralization carbon capture and storage. *Catalysis Science & Technology*.

[15] Power IM, Harrison AL, Dipple GM, Southam G (2013). Carbon sequestration via carbonic anhydrase facilitated magnesium carbonate precipitation. *International Journal of Greenhouse Gas Control*.

[16] Bhaduri GA, Henderson RA, Šiller L Reply to the ‘Comment on “Nickel nanoparticles catalyse reversible hydration of carbon dioxide for mineralization carbon capture and storage”’ by D. Britt, *Catalysis Science & Technology*, 2013, 3. *Catalysis Science & Technology*.

[17] Soong Y, Fauth DL, Howard BH (2006). CO_2_ sequestration with brine solution and fly ashes. *Energy Conversion and Management*.

[18] Liu Q, Maroto-Valer MM (2012). Studies of pH buffer systems to promote carbonate formation for CO_2_ sequestration in brines. *Fuel Processing Technology*.

[19] Santos RM, Ceulemans P, Van Gerven T (2012). Synthesis of pure aragonite by sonochemical mineral carbonation. *Chemical Engineering Research and Design*.

[20] Bodor M, Santos RM, Kriskova L, Elsen J, Vlad M, Van Gerven T (2013). Susceptibility of mineral phases of steel slags towards carbonation: mineralogical, morphological and chemical assessment. *European Journal of Mineralogy*.

